# P38 MAPK and glucocorticoid receptor crosstalk in bronchial epithelial cells

**DOI:** 10.1007/s00109-020-01873-3

**Published:** 2020-01-23

**Authors:** Simon Lea, Jian Li, Jonathan Plumb, Kate Gaffey, Sarah Mason, Rosie Gaskell, Chris Harbron, Dave Singh

**Affiliations:** 1 University of Manchester, NIHR Translational Research Facility, University Hospital of South Manchester, Manchester, M23 9LT UK; 2 Roche Pharmaceuticals, 6 Falcon Way, Welwyn Garden City, AL7 1TW UK

**Keywords:** Corticosteroid insensitivity, Glucocorticoid receptor, p38 MAPK, Asthma, Aronchial epithelial cells

## Abstract

**Abstract:**

p38 MAPK inhibition may have additive and synergistic anti-inflammatory effects when used with corticosteroids. We investigated crosstalk between p38 MAPK inhibitors and corticosteroids in bronchial epithelial cells to investigate synergistic effects on cytokine production and the molecular mechanisms involved. Effects of the p38 MAPK inhibitor BIRB-796 and dexamethasone alone and in combination on LPS, polyI:C or TNFα -induced IL-6, CXCL8 and RANTES were assessed in 16HBEs (human epithelial cell line) and on TNFα-induced IL-6 and CXCL8 in primary human epithelial cells from asthma patients and healthy controls. 16HBEs were used to assess effects of BIRB-796 alone and in combination with dexamethasone on glucocorticoid receptor (GR) activity by reporter gene assay, expression of GR target genes and nuclear localisation using Western blot. The effects of BIRB-796 on TNFα stimulated phosphorylation of p38 MAPK and GR at serine (S) 226 by Western blot. Epithelial levels of phosphorylated p38 MAPK and GR S226 were determined by immunohistochemistry in bronchial biopsies from asthma patients and healthy controls. BIRB-796 in combination with dexamethasone increased inhibition of cytokine production in a synergistic manner. Combination treatment significantly increased GR nuclear localisation compared to dexamethasone alone. BIRB-796 inhibited TNFα-induced p38 MAPK and GR S226 phosphorylation. Phosphorylated GR S226 and p38 MAPK levels were increased in bronchial epithelium of more severe asthma patients. Molecular crosstalk exists between p38 MAPK activation and GR function in human bronchial epithelial cells, which alters GR activity. Combining a p38 MAPK inhibitor and a corticosteroid may demonstrate therapeutic potential in severe asthma**.**

**Key messages:**

• Combination of corticosteroid and p38 inhibitor in human bronchial epithelial cells

• Combination increased cytokine inhibition synergistically and nuclear GR

• p38 MAPK inhibition reduced TNFα-induced phosphorylation of GR at S226 but not S211

• Phosphorylated GRS226 and p38 is increased in bronchial epithelium in severe asthma

• Combining a p38 inhibitor and a corticosteroid may be effective in asthma treatment

**Electronic supplementary material:**

The online version of this article (10.1007/s00109-020-01873-3) contains supplementary material, which is available to authorized users.

## Introduction

Inhaled corticosteroids (ICS) are the mainstay of anti-inflammatory treatment for asthma. However, many asthma patients treated with ICS have suboptimal asthma control [1], highlighting the need for novel anti-inflammatory treatments.

The glucocorticoid receptor (GR) is a ligand-dependent transcription factor that resides in the cytoplasm until ligand binding causes nuclear translocation, resulting in transrepression or transactivation of gene transcription [2]. There are numerous phosphorylation sites within the GR N-terminus, changes in which can lead to the alterations of GR function through ligand binding, nuclear localisation, modulating interactions with co-regulators or transcriptional activation [3, 4]. Two such phosphorylation sites are serine (S) 211 and S226 which have roles in subcellular localisation [3]; GR-ligand nuclear translocation is associated with phosphorylation of S211, while GR nuclear export is associated with S226 phosphorylation.

p38 mitogen-activated protein kinase (MAPK) signalling promotes the secretion of inflammatory proteins through transcription factor activation [5], post-transcriptional mRNA stabilization and enhancement of protein translation [6, 7]. p38 MAPK inhibitors suppress the inflammatory responses of various cell types within the lung, including alveolar macrophages, lung lymphocytes, airway smooth muscle cells and bronchial epithelial cells [8–10]. Combining p38 MAPK inhibitors and corticosteroids results in additive anti-inflammatory effects in these cell models, due to the different mechanisms of action [8, 11]. Furthermore, these studies have also shown some evidence that the combination effects are more than additive, with a synergistic interaction observed [8, 11].

p38 MAPK can phosphorylate GR serine residues, altering ligand affinity and transcriptional activity [12–14]. It has been reported that p38 MAPK activation reduces S211 phosphorylation [15, 16], thereby decreasing GR activity. However, p38 MAPK regulation of GR phosphorylation is cell specific, as the effect on S211 phosphorylation is not present in all cell models [17, 18]. Peripheral blood mononuclear cells (PBMCs) from severe asthma patients show a reduction of nuclear GR, which is associated with increased GR S226 phosphorylation [19]. A possible mechanism for the synergistic interaction between p38 MAPK inhibitors and corticosteroids is via regulation of either GR S211 or S226 phosphorylation to enhance GR nuclear localisation.

Epithelial cells play a central role in the pathophysiology of asthma through the release of pro-inflammatory mediators in response to environmental triggers such as infection or stimulation with cytokines [20]. p38 MAPK activation is increased in alveolar macrophages, airway epithelial cells and airway smooth muscle cells of severe asthma patients compared with controls [21–23]. p38 MAPK inhibition of the inflammatory responses of epithelial cells in severe asthma may have therapeutic potential, particularly if there is a synergistic interaction with corticosteroids.

The aim of this study was to investigate crosstalk between p38 MAPK inhibitors and corticosteroids in bronchial epithelial cells. We used a human bronchial epithelial cell (HBEC) line to investigate whether the anti-inflammatory interactions between these drug classes on cytokine production were additive or synergistic. We also investigated whether p38 MAPK inhibition altered GR function by measuring GR activity, nuclear localisation and phosphorylation. Additionally, we used lung samples from asthma patients and healthy controls to study the combination anti-inflammatory effects of these drugs on bronchial epithelial cells, and to perform immunohistochemical analysis of bronchial epithelial cells to further investigate the relationship between p38 MAPK activation and GR phosphorylation.

## Methods

### Human bronchial epithelial cell culture

The human bronchial epithelial cell line 16HBE14o^−^ (ATCC; HBEC) was maintained in supplemented Minimal Essential Medium Eagle (MEME, Gibco, Fisher Scientific UK Ltd., Loughborough, UK). Confluent cells were grown in tissue culture plates until confluent and then incubated in fresh supplemented media in the presence of either lipopolysaccharide (LPS) (1 μg/ml, Sigma, Poole, UK), polyinosinic:polycytidylic acid (poly I:C) (100 μg/ml, Source Bioscience, Nottingham, UK) or tumour necrosis factor (TNFα) (10 ng/ml, Peprotech, London, UK). Cells were pre-incubated with dexamethasone (0.1–1000 nM, Sigma) and/or the p38 MAPK inhibitor BIRB-796 (0.1–1000 nM, Sigma) and incubated in 5% CO_2_ at 37 °C for varying time points. Plates were centrifuged at 2000 rpm for 10 min at 4°C and cell-free supernatants removed and stored at −80°C for cytokine analysis.

To assess the effects of LPS, poly I:C and TNFα stimulation on phosphorylated p38 MAPK expression, HBECs were stimulated with LPS (1 μg/ml) or poly I:C (100 μg/ml) for 0–180 min or with TNFα (10 ng/ml) for 0–60 min.

To assess the effects of dexamethasone and BIRB-796 on stimulated phosphorylated p38 MAPK expression, HBECs were pre-incubated for 60 min with either dexamethasone (1000 nM) , BIRB-796 alone (1000 nM) or both in combination, followed by stimulation with LPS (1 μg/ml) or poly I:C (100 μg/ml) for 60 min or with TNFα (10 ng/ml) for 15 min (based on preliminary time course experiments; Fig. S1).

To assess the effects of dexamethasone and BIRB-796 on GR target gene expression, HBECs were cultured with or without TNFα (10 ng/ml) for 15 min followed by dexamethasone (100 nM) and BIRB-796 (100 nM) both alone and in combination for 4 h.

To investigate the effects of dexamethasone on GR phosphorylation at S211 and S226 residues, HBECs were incubated with dexamethasone (1000 nM) or left untreated for 15–180 min.

To assess whether TNFα-induced phosphorylation of GR S226 is p38 MAPK-dependent, HBECs were stimulated with TNFα (10 ng/ml) for 15–180 min with and without pre-incubation of BIRB-796 (1000 nM) for 60 min.

### Study subjects

Patients with a diagnosis of asthma (*n* = 23) and healthy control subjects (*n* = 15) were recruited; all subjects were required to be lifelong non-smokers. Patients performed spirometry for measurement of forced expiratory volume in 1 s (FEV_1_) and reversibility to inhaled salbutamol (200 μg), asthma control questionnaire (ACQ), and skin prick testing using house dust mite, cat and grass allergens (Soluprick SQ, Alk Abelló (UK) Ltd). The study was approved by the local research ethics committee (NRES Committee North West – Greater Manchester South; REC Ref: 06/Q1403/156). All subjects provided written informed consent.

### Bronchoscopy

Patients with differing asthma severity as defined by the Global Initiative for Asthma (GINA) score (GINA 1, *n* = 5; GINA 2, *n* = 7; and GINA 3/4, *n* = 6) and healthy control subjects (*n* = 10) underwent bronchoscopy to obtain bronchial biopsies from the lower lobes; these were formalin-fixed and paraffin-embedded as previously described [24]. Asthma patients (*n* = 5) and healthy control subjects (n = 5) underwent bronchoscopy to obtain brushings from the lower lobe segmental airways.

### Primary epithelial cell culture

Bronchial epithelial cells obtained from asthma patients and healthy controls were gently removed from the airway by brushing with a cytology brush (width 5 mm, length 10 mm) (Olympus, Southend-on-sea, UK) across the surface of the airway wall. Cells were then placed into fully supplemented BEBM Media (BEBM bulletkit Lonza, Slough, UK) on ice by passing the sheath over the head of the brush to dislodge cells. Cells were centrifuged (400 g, 10 min, 4°C) before resuspending into supplemented BEBM media. The whole cell suspension was seeded into collagen-coated (Nutacon, Leimuiden, Netherlands) flasks. Cells were left to adhere and grow and media was replaced twice weekly. When confluent, cells were passaged and plated out into 96-well plates. When 80% confluence was reached, cells were pre-incubated for 60 min with either dexamethasone (100 nM), BIRB-796 (100 nM) or both drugs in combination before stimulation with TNFα (10 ng/ml) for 24 h.

### Cytokine measurements

Enzyme-linked immunosorbent assays (ELISA) were used to determine the supernatant level of Interleukin (IL)-6, The chemokine (C-X-C motif) ligand 8 (CXCL8), and Regulated on Activation, Normal T Cell Expressed and Secreted (RANTES) according to the manufacturer’s instructions (R&D Systems, Abingdon, UK). Lower limits of quantification were 32.5 pg/ml for CXCL8 and RANTES and 9.375 pg/ml for IL-6.

### Western blot

Western blot analysis was performed using the following antibodies: phospho-p38 MAPK (New England Biolabs, Hitchin, UK), total p38 MAPK (New England Biolabs), phospho-GR S226 (Abcam, Cambridge, UK), phospho-GR S211 (New England Biolabs), total GR (BD bioscience), β-actin (Abcam) and histone-3 (Cell Signalling Technology, MA, USA) (for full details, see the online supplement). All Western blot protein molecular weights are shown in Fig. S2a. HBECs were lysed for either whole cell lysate or cellular fractions (see online supplement). Nuclear and cytoplasmic fractions were confirmed by the presence of nuclear protein histone-3 and cytoplasmic protein β-actin, respectively. The absence of histone-3 in the cytoplasmic fractions and absence of β-actin in nuclear fractions were confirmed in *n* = 5 separate cultures (Fig. S2b).

### GR- reporter gene assay

16HBE14o- cells were stably transfected with pGL44.36[luc2P/MMTV/Hygro] vector (Promega) (SalI linearized) using FuGENE6 (Roche, Germany) (as described in online supplement).

Cells with luciferase reporters were treated with dexamethasone, BIRB-796, SB239063, budesonide and TNFα, either individually or in combination. Bioluminescence signals were analysed in BRASS software and RAP algorithm. Data were presented as photon counts per minute (cpm).

### GR target gene PCR

RNA was obtained as described in the online supplement. Gene expression was analysed using TaqMan gene expression assays for GLIZ, FKBP5 and the endogenous control glyceraldehyde-3-phosphate dehydrogenase (GAPDH). Relative expression levels compared to unstimulated controls were determined using the 2^-ΔΔCt^ method. Full details are described in online supplement.

### Immunohistochemistry

Bronchial biopsies were stained with rabbit antihuman GR S226 (Abcam, Cambridge, UK) and rabbit antihuman phosphorylated p38 MAPK (Cell Signalling Technology) coupled with an ImmPRESS™ Excel Amplified HRP Polymer Staining Kit (Anti-Rabbit IgG) with 3,3′ diaminobenzidine as a substrate (Vector Laboratories, CA, USA). Sections were counterstained in Gills haematoxylin (for full details, see the online supplement).

Images were captured using a Nikon Eclipse 80i microscope (Nikon UK Ltd) with an attached QImaging digital camera (Media Cybernetics, MD, USA). Percentages of GR S226- and phosphorylated p38 MAPK-positive epithelial cells were calculated.

### Data analysis

The data and statistical analyses comply with the recommendations on experimental design and analysis in pharmacology [25].

One-way analysis of variance (ANOVA) followed by Tukey-Kramer multiple comparisons tests was used for ELISA and immunohistochemistry data to compare between conditions and subject groups. Pearson’s rank was used to assess correlations between phosphorylated p38 MAPK and GR S226 expression. Repeated measures ANOVA with Dunnett multiple comparisons post-test were used to analyse immunofluorescence and Western blot analysis. Maximal inhibition was defined as the effect of the drug at the highest concentration. IC_50_ values were determined using a four-parameter non-linear iterative curve fitting analysis. A dose-sparing analysis to assess whether equivalent responses can be achieved at lower doses of compound than expected given the monotherapy responses and an efficacy-enhancing analysis to assess whether the combination results in a significantly greater maximal effect than either compound as monotherapies were performed [26] and described in full in the online supplement. To assess whether combination treatment in primary epithelial experiments had an additive or synergistic effect, an interaction ratio (IR) was calculated based on observed and expected inhibitions [27]. All statistical analysis was performed in GraphPad Prism (GraphPad Software, http://www.graphpad.com). *P* < 0.05 was considered significant.

## Results

### Inhibition of cytokine production from HBECs

The stimulation of HBECs by LPS, poly I:C and TNFα significantly increased secretion of IL-6, CXCL8 and RANTES; (*p* < 0.05 for all comparisons of stimulated against unstimulated levels; Fig. S3). LPS-, poly I:C- and TNFα-stimulated cytokine secretion was significantly reduced by dexamethasone or BIRB-796 in a concentration-dependent manner (Fig. S3). Differences in the magnitude of maximal inhibition (i.e., inhibition at 1000 nM) between cytokines were observed. For example, the maximal inhibition of LPS- and TNFα-stimulated IL-6 production was greater than RANTES and CXCL8 secretion (*p* < 0.05 for all comparisons; described further in the online supplement and in Table S1). The maximal inhibition caused by BIRB-796 and dexamethasone was generally similar, with any differences described online (Table S1).

**Fig. 1:**
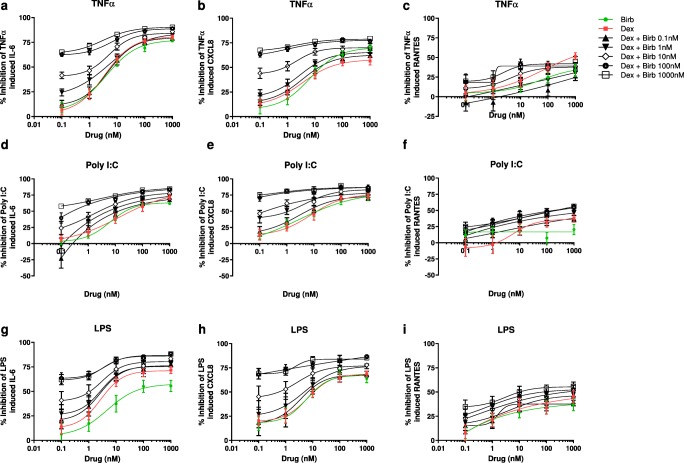
Combination effect of dexamethasone and BIRB-796 on LPS-, poly I:C- or TNFα-induced cytokines in human bronchial epithelial cells. 16HBE14o− cells were pre-treated with dexamethasone (0.1–1000 nM), BIRB-796 (0.1–1000 nM) either alone or in combination at all concentrations or with vehicle (DMSO 0.05%) for 1 h before 24 h stimulation with either TNFα (10 ng/ml) (**a**-**c**), poly I:C (100 μg/ml) (**d**-**e**) or LPS (1μg/ml) (**g**-**i**). Supernatants were collected and assayed for IL-6 (**a**, **d** and **g**), CXCL8 (**b**, **e** and **h**) or RANTES (**c**, **f** and **i**) release by ELISA (*n* = 12 separate cultures). Data presented as mean ± SEM percentage inhibition of -induced IL-6, CXCL8 or RANTES. Lines represent four-parameter non-linear dose response curves

Using BIRB-796 in combination with dexamethasone generally provided greater inhibition of LPS-, poly I:C- or TNFα-induced cytokine production compared with either drug alone (Fig. 1a-i. The maximum inhibition of cytokine production using the highest concentration of dexamethasone in combination with BIRB-796 was significantly greater compared to either drug alone for many experimental conditions (analysis presented in Table S2).

**Fig. 2:**
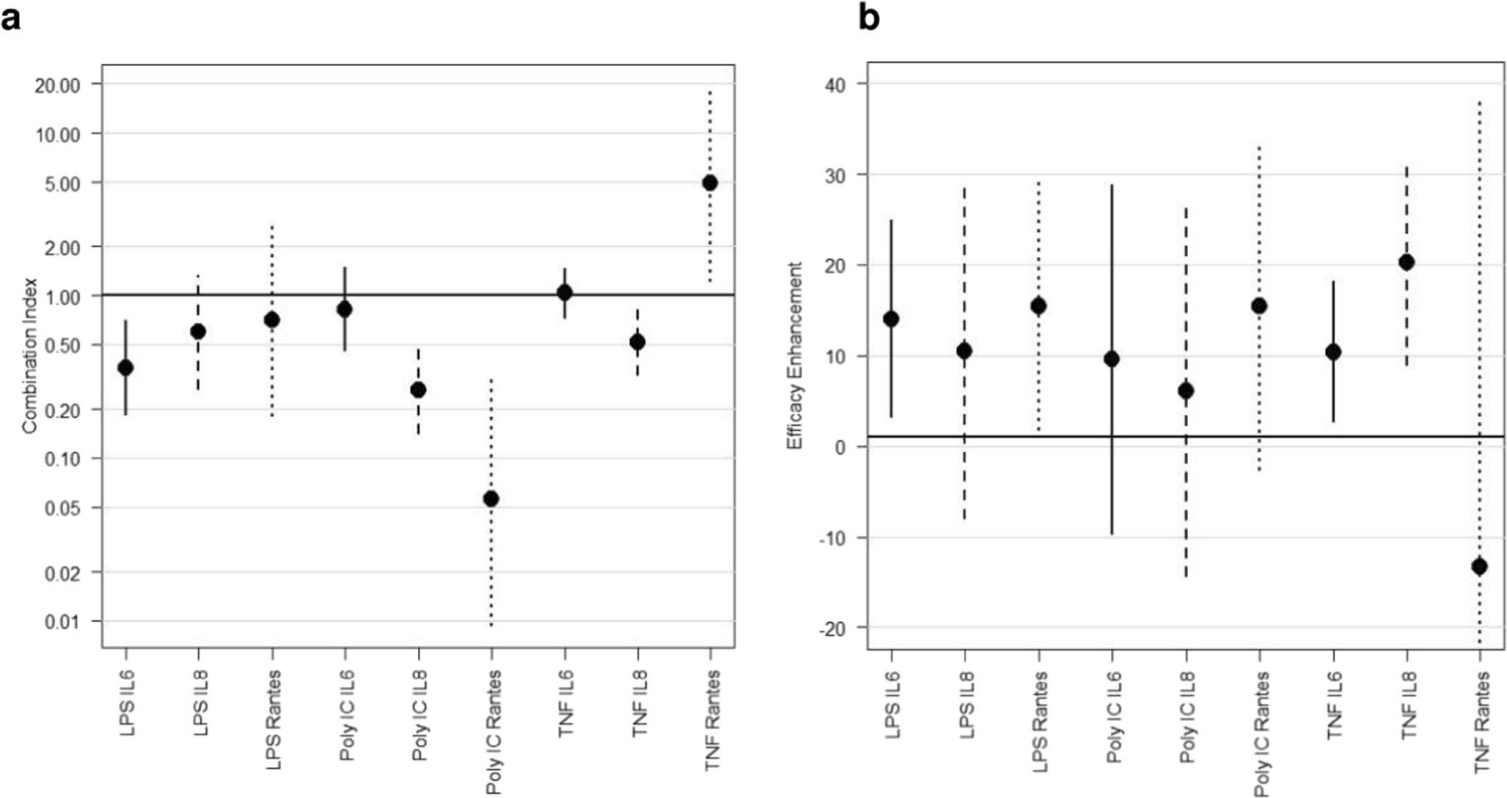
Combination effect of dexamethasone and BIRB-796: dose-sparing and efficacy-enhancing analysis. Dose-sparing analysis to assess whether equivalent responses can be achieved at lower doses of compound than expected given the monotherapy responses (**a**) and an efficacy-enhancing analysis to assess whether the combination results in a significantly greater maximal effect than either compound as monotherapies (**b**) were performed. Estimates of the combination index (**a**) or efficacy-enhancing benefit (**b**) are plotted with 95% confidence intervals calculated for each stimulus and cytokine. A combination index of one (**a**) or efficacy-enhancing benefit of zero (**b**) corresponds to additivity. Endpoints where the 95% confidence interval lies completely below one (**a**) or above zero (**b**) show statistically significant synergy

Figure 2a shows dose-sparing analysis to assess whether equivalent responses can be achieved at lower doses of compound than expected given the monotherapy responses. A dose-sparing combination index where the entirety of the 95% confidence interval lies below one (Fig. 2a) indicates a significant synergistic effect. Figure 2b shows an efficacy-enhancing analysis to assess whether the combination results in a significantly greater maximal effect than either compound as monotherapies. An efficacy-enhancing benefit where the entirety of the 95% confidence interval lies above zero (Fig. 2b) indicates a significant synergistic effect. A combination treatment showed a significant synergistic dose-sparing effect on LPS-induced IL-6, poly I:C-induced CXCL8 and RANTES and TNFα-induced CXCL8. There was a significant synergistic efficacy-enhancing benefit for LPS-induced IL-6 and RANTES and TNFα-induced IL-6 and CXCL8.

We next investigated whether these synergistic effects were due to p38 MAPK modulation of glucocorticoid receptor activity. Therefore, we investigated the effects of p38 MAPK inhibition on GR activity, cellular location and phosphorylation status.

### Effects of p38 MAPK inhibition on corticosteroid-induced GR reporter activity

**Fig. 3:**
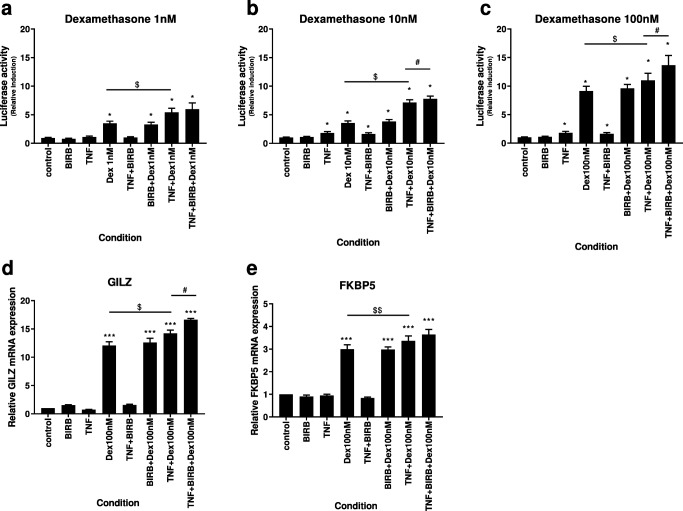
Effects of p38 MAPK inhibition on dexamethasone-induced GR reporter activity and GR target genes. 16HBE14o− cells transfected with GR-luciferase reporter (**a**-**c**) or left alone (**d** and **e**) were stimulated with TNF-α (10 ng/ml) or left unstimulated followed by treatment with dexamethasone (1–100 nM (**a**-**c**, 100 nM **d**-**e**), BIRB-796 (1000 nM) alone and in combination with dexamethasone at all concentrations (**a**-**e**). GR-reporter activity was assessed by luminescence (**a**-**c**) or relative expression of GILZ (**d**) and FKBP5 (**e**) by real-time q-PCR. Data represent mean ± SEM of relative activity (a-c), fold change expression (**d** and **e**) compared to basal levels from *n* = 6 (**a**-**c**) or *n* = 5 (**d** and **e**) experiments. *, *** = significantly above basal control (*p* < 0.05, 0.001). # = significantly increased above dexamethasone alone in either stimulated or unstimulated cells (*p* < 0.05). $, $ = significantly increased above dexamethasone alone in unstimulated cells (*p* < 0.05, 0.01)

Dexamethasone (1–100 nM) and budesonide (1–100 nM) significantly induced GR-reporter activity above the untreated controls (*p* < 0.05) (Fig. 3a-c and Fig. S4, respectively). There was no effect of BIRB-796 in combination with either corticosteroid except for budesonide 1 nM combined with BIRB-796 (1000 nM) which significantly increased GR-reporter activity above budesonide alone (*p* < 0.05) (Fig. 3e). SB239063 (1000 nM) had no effect in combination with dexamethasone (Fig. S4).

TNFα (10 ng/ml) stimulation caused a small increase in GR-reporter activity. Dexamethasone (1–100 nM) and budesonide (1–100 nM) caused large increases in GR-reporter activity in TNFα-stimulated cells (*p* < 0.05 for all comparisons), with the addition of BIRB-796 or SB239063 causing further significant upregulation of GR-reporter activity compared to corticosteroid alone (p < 0.05 for all comparisons).

### Effects of p38 MAPK inhibition on corticosteroid-induced GR target genes

To determine if increased GR activity observed translated to increased induction of GR target genes, the effects of combination treatment of BIRB-796 and dexamethasone on GILZ and FKBP5 were assessed.

Dexamethasone (100 nM) significantly induced GILZ and FKBP5 expression above the level of untreated controls (*p* < 0.05) (Fig. 3d and e). There was no effect of BIRB-796 in combination with dexamethasone.

TNFα (10 ng/ml) stimulation or BIRB-796 treatment alone had no effect on GLIZ or FKBP5 expression. Dexamethasone (100 nM) also caused increases in GILZ and FKBP5 expression in TNFα stimulated cells (*p* < 0.05 for all comparisons), with the addition of BIRB-796 causing further significant upregulation of GILZ expression compared to corticosteroid alone (*p* < 0.05 for all comparisons).

### GR nuclear localisation

**Fig. 4:**
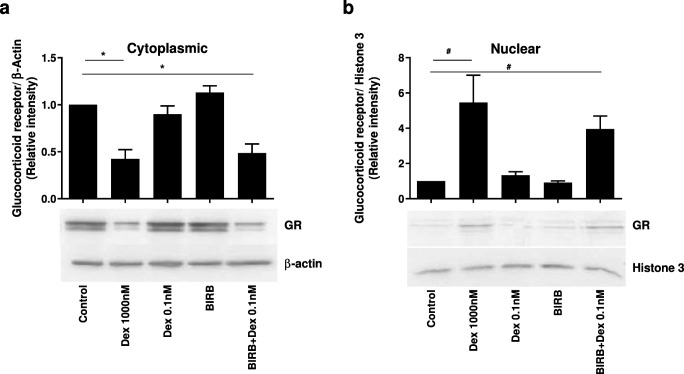
Effect of BIRB-796 on dexamethasone-induced nuclear localisation of glucocorticoid receptor. 16HBE14o− cells were treated with dexamethasone alone (0.1 and 1000 nM), BIRB-796 alone (1000 nM) or dexamethasone (0.1 nM) in combination with BIRB-796 (1000 nM). Cytoplasmic (**a**) and nuclear (**b**) cellular fractions were obtained and analysed for GR levels by Western blot analysis. Band density was normalised to β-actin for cytoplasmic or histone-3 for nuclear fractions. Representative blots are shown under corresponding conditions. Data presented as mean ± SEM (*n* = 5). * = significantly below basal control in cytoplasmic fraction (*p* < 0.05). # = significantly above basal control in nuclear fraction (*p* < 0.05)

The effects of p38 MAPK inhibition on GR cellular location were investigated by Western blot analysis, which confirmed that dexamethasone (1000 nM) significantly reduced GR cytoplasmic protein levels and significantly increased nuclear GR levels compared with untreated controls (*p* < 0.05 for both comparisons; Fig. 4). Dexamethasone (0.1 nM) alone and BIRB-796 (1000 nM) alone had no effect on GR levels in either cellular compartment compared with untreated controls. Combining dexamethasone (0.1 nM) with BIRB-796 (1000 nM) reduced cytoplasmic GR and significantly increased nuclear GR levels compared with untreated controls (*p* < 0.05 both comparisons) (Fig. 4).

### Effects of p38 MAPK inhibition on corticosteroid-induced GR phosphorylation

**Fig. 5:**
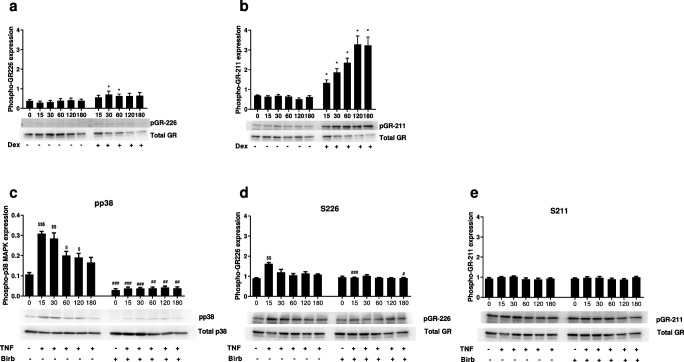
Effect of dexamethasone or TNFα with/without p38 MAPK inhibition on phosphorylation of p38 MAPK and GR at S226 and S211. 16HBE14o− cells were stimulated with dexamethasone (1000 nM) alone (**a** and **b**) or TNFα with/without BIRB-796 (1000 nM) for 15, 30, 60, 120 and 180 min. Cells were lysed and assessed for phosphorylation of p38 MAPK (**c**), GR S226 (**a** and **d**) or GR S211 (**b** and **e**) by Western blotting. Band density was normalised to total GR (**a**, **b**, **d** and **e**) or total p38 MAPK (**c**). Representative blots are shown under corresponding conditions. Data presented as mean ± SEM (*n* = 5). * = significantly above time-matched untreated control (*p* < 0.05). $, $$, $$$ = significantly above untreated control (*p* < 0.05, 0.01, 0.001). #, ##, ### = significantly below time-matched TNFα only treatment **(***p* < 0.05, 0.01, 0.001)

The effects of p38 MAPK inhibition on GR phosphorylation status were investigated by Western blot. The phosphorylation of GR S226 and GR S211 by dexamethasone administered alone was confirmed, with a relatively larger increase in GRS211 phosphorylation observed (Fig. 5a and b respectively).

LPS, poly I:C or TNFα caused phosphorylation of p38 MAPK in HBECs, which was inhibited by BIRB-796 but not by dexamethasone (Fig. S5). TNFα was used to further investigate the effects of BIRB-796 inhibition on p38 MAPK and GR phosphorylation. TNFα stimulation for 15 min significantly increased phosphorylation of p38 MAPK (15–120 min) and GR S226 above basal levels but had no effect on GR S211 phosphorylation (Fig. 5c-e). Pre-incubation with BIRB-796 significantly reduced basal and TNFα-induced (15–180 min) p38 MAPK phosphorylation. Pre-incubation with BIRB-796 also significantly reduced TNFα-induced (15 mins) GR S226 phosphorylation (Fig. 5c and d, respectively) but had no effect on phosphorylated GR S211 levels (Fig. 5e).

### Combination effects of dexamethasone and BIRB-796 in primary bronchial epithelial cells

Next, we sought to confirm our findings using primary bronchial epithelial cells from both asthma patients and control subjects. Two of the five asthma patients were treated with inhaled corticosteroids. The mean FEV_1_ was 72.8% predicted with mean ACQ 1.74, indicating suboptimal asthma control; see Table 1 for demographics.

**Table 1:** Subject demography

	Immunohistochemistry					Bronchial brushes		
Clinical characteristics	Healthy (*n* = 10)	GINA 1 (*n* = 5)	GINA 2 (*n* = 7)	GINA 3/4 (*n* = 6)	*p* value	Healthy (n = 5)	Asthma (n = 5)	P value
Gender Male/Female	5/5	4/1	4/3	4/6		4/1	4/1	
Age	27 (20–37)	48 (41–63)	41 (33–68)	39 (22–60)	*p* > 0.05	44 (40–46)	39 (25–65)	p > 0.05
ICS users	0/10	0/6	7/7	6/6		0/5	2/5	
Daily ICS dose^a^ (mcg)	0 (0)	0 (0)	200 (0–400)	500 (500–2000)	*p* < 0.001	0 ± 0	300 ± 141	*p* < 0.01
LABA users	0/10	0/6	0/7	6/6		0/5	0/5	
Atopy	0/10	4/6	4/7	4/6		0/5	5/5	
FEV_1_% predicted (%)	97 ± 10.4	87.7 ± 8.9	71.6 ± 15.4	71.8 ± 23.6	p < 0.001	110 ± 9.6	72.8 ± 11.0	p < 0.001
Reversibility (mls)		273 ± 145	411 ± 232	410 ± 348	p < 0.05	140 ± 60	682 ± 438	p < 0.01
Reversibility (%)	4.78 ± 2.12	9.45 ± 6.58	19.3 ± 14.39	21.16 ± 13.52	p < 0.05	3.48 ± 1.72	26.0 ± 18.8	p < 0.01
ACQ-7 Score	N/A	0.86 ± 0.15	1.44 ± 0.76	2.0 ± 0.99	*p* < 0.05	N/A	1.74 ± 0.48	

Data are presented as means ± SD, median (range) for age. Asthma control questionnaire (ACQ), forced expiratory volume (FEV), inhaled corticosteroids (ICS), long acting beta agonists (LABA)

^a^Beclometasone equivalent dose

**Fig. 6:**
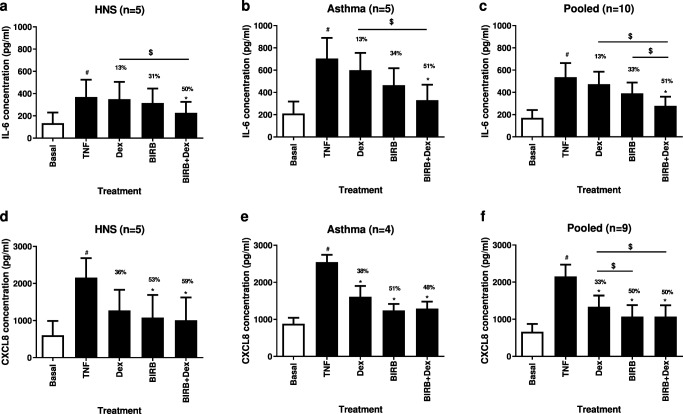
Combination effect of dexamethasone and BIRB-796 on TNFα-induced cytokines in primary human bronchial epithelial cells. Primary bronchial epithelial cells from healthy subjects (HNS) (*n* = 5) or patients with asthma (*n* = 5) were pre-treated with dexamethasone (100 nM) and BIRB-796 (100 nM) alone or in combination or with vehicle (DMSO 0.05%) for 1 h before 24-h stimulation with TNFα (10 ng/ml) or media (Basal). Supernatants were collected and assayed for IL-6 (**a**-**c**) or CXCL8 (**d**-**f**) release by ELISA. Data presented as mean ± SEM TNFα induced cytokine levels with percentage inhibition shown above each bar. # = significantly increased above basal levels (*p* < 0.05). * = significantly below TNFα-stimulated vehicle control (*p* < 0.05). $ = significantly increased inhibition above dexamethasone or BIRB-796 alone (*p* < 0.05)

TNFα induced a robust cytokine response in HBECs and so was chosen as the stimulus for primary bronchial epithelial cells; TNFα significantly increased secretion of IL-6 and CXCL8 from both healthy subjects and asthma patients (*p* < 0.05 for all comparisons; Fig. 6). Dexamethasone (100 nM) alone and BIRB-796 (100 nM) alone did not significantly inhibit IL-6 release in either asthma patients or controls (Fig. 6). Dexamethasone (100 nM) alone significantly inhibited CXCL8 in asthma patients, BIRB-796 (100 nM) alone significantly inhibited CXCL8 in both asthma patients and controls. Dexamethasone in combination with BIRB-796 caused significant inhibition of IL-6 and CXCL8 in both asthma patients and controls (*p* < 0.05 both comparisons) with inhibition significantly increased above dexamethasone alone in both groups for IL-6 but not CXCL8.

As no differences in drug effects were observed between subject groups (Fig. S6), data were pooled to give additional power for an exploratory IR analysis (Fig. 6c and f). Combination treatment provided significantly greater inhibition of IL-6 than observed with either drug alone (*p* < 0.05). IR calculation for the combination of dexamethasone and BIRB-796 predicted a 34.5% inhibition of IL-6 due to additive anti-inflammatory effects, while the observed effect was 44.3% with an IR of 1.28, falling short of the 1.5 required to demonstrate a synergistic interaction. BIRB-796 alone and in combination with dexamethasone caused significant CXCL8 inhibition that was similar in magnitude.

### Expression of phosphorylated p38 MAPK and GR S226 in bronchial epithelium of asthma

**Fig. 7:**
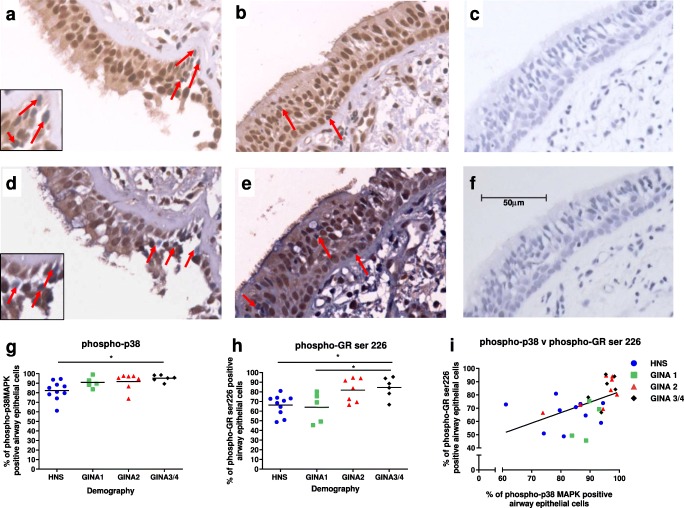
Expression of phosphorylated p38 MAPK and GR S226 in the bronchial epithelium of patients with asthma and healthy controls. Expression of phosphorylated p38 MAPK and GR at S226 was examined by immunohistochemistry in bronchial biopsy tissue from healthy subjects (*n* = 10) and asthma patients of differing severity (GINA 1, *n* = 5; GINA 2, *n* = 7; GINA 3/4, *n* = 6). Representative images are shown for healthy subjects (HNS) (**a** and **d**) and GINA3/4 (**b** and **e**) for phospho-p38 MAPK (**a** and **b**) and phosphor- GR S226 (**d** and **e**), negative controls for p38 MAPK and GR S226 (**c** and **f**, respectively). Red arrows indicate negative cells within epithelium. Data presented as positive cells per mm of epithelium for phospho-p38 MAPK (**g**) and phospho- GR S226 (**h**) and both (**i**). Data represents individual patients with mean (**g** and **h**) and linear regression (**i**). * = significant between groups (*p* < 0.05)

Bronchial biopsies from healthy controls and asthma patients were analysed. Asthma patients were categorized as GINA 1 (no ICS use), GINA 2 (ICS low dose) and GINA 3/4 (ICS plus long-acting beta agonist treatment); demographics are shown in Table 1. The percentage of bronchial epithelial cells positively stained for both phosphorylated p38 MAPK and phosphorylated GR at S226 was significantly higher in GINA 3/4 asthma patients compared with healthy controls (*p* < 0.05 both comparisons; Fig. 7g and h, respectively) and compared with GINA 1 for GR S226 (*p* < 0.05). There was no significant difference in the percentage of epithelial staining between healthy controls and GINA 1 or GINA 2 for either phosphorylated p38 MAPK or GR. There was also a significant correlation between percentage positive staining for phosphorylated p38 MAPK and GR S226 in the whole study population (*R*^*2*^ = 0.25, *p* < 0.05; Fig. 7i).

## Discussion

Combining a corticosteroid and a p38 MAPK inhibitor in a HBEC line caused increased inhibition of inflammatory mediator release in a synergistic manner for both dose-sparing and efficacy-enhancing effects. This synergy was observed in some, but not all, experimental conditions, varying with the stimulus used and cytokine measured. GR reporter and GR target gene experiments demonstrated an enhancement of GR activity by p38 MAPK inhibition in TNFα-stimulated epithelial cells. Furthermore, p38 MAPK inhibition enhanced corticosteroid-induced GR nuclear localisation. Previous studies have investigated the crosstalk between p38 MAPK and GR function [8, 15, 16, 19], showing that the nature of this crosstalk appears to vary between cell types, but the mechanism of crosstalk in bronchial epithelial cells has not been studied. Focusing on human bronchial epithelial cells, our key findings are that p38 MAPK inhibition enhances GR function, demonstrated through inhibition of inflammatory cytokines, GR reporter activity, GR target gene experiments and GR nuclear localisation.

We observed that p38 MAPK inhibition reduced TNFα-induced phosphorylation of GR at S226 but not S211. We also demonstrated GINA 3/4 asthma patients had higher expression levels of both p38 MAPK and GR S226 phosphorylation in the bronchial epithelium compared with mild asthma patients and healthy controls. High bronchial epithelial p38 MAPK phosphorylation levels have been previously reported in severe asthma [23]; We have confirmed these findings and show an association with phosphorylated GR S226 expression levels. This association in asthma patients, together with our experiments that demonstrated a role for p38 MAPK in GR S226 phosphorylation, implicates p38 MAPK activation as a mechanism that can modulate GR activity in asthma.

The downstream implications of the increased phosphorylated p38 MAPK observed in asthma epithelium were investigated by the GR reporter assay and GR target gene experiments to determine the effects of p38 MAPK on GR activity. TNFα stimulation activated p38 MAPK; pharmacological inhibition of this TNFα-induced p38 MAPK activation led to enhanced GR activity. This effect was confirmed using different corticosteroids and p38 MAPK inhibitors. While the use of these additional drugs were limited to the GR reporter assay, it would be of interest to investigate their effects in other assays, for example, including the effects of budesonide in combination with a p38 MAPK inhibitor in primary cells. However, our data suggest that the crosstalk observed is a class effect.

Our data show that the GR reporter responds to TNFα alone. The reporter used is widely accepted as a reporter for GR signalling [28, 29]; however, the promoter can be activated by androgen receptor (AR) as well as glucocorticoid receptor. TNFα can induce AR expression, and there is a crosstalk between NFkβ and AR signalling at genome level in mammalian cells [30, 31]. It is possible that the reporter responded to TNFα signalling through AR and not GR. The level of induction of the reporter activity by TNFα alone is however much smaller (10%–15%) compared to that induced by dexamethasone (approximately 500%). Also, we did not observe an increase in the expression of the GR target genes GILZ or FKBP5 above controls in response to TNFα alone.

Corticosteroid and p38 MAPK inhibitor effects on cytokine production from HBECs varied according to the stimulant used and the cytokine measured. Such differences have also been observed in PBMCs and alveolar macrophages [8, 11, 32–36]. This is due to differences in the signalling pathways activated by different stimuli; these pathways may show varying degrees of sensitivity to different drugs. The effects of high corticosteroid concentrations often provided only approximately 50%–70% inhibition of cytokine production. Consistent with our data, other publications, using epithelial cell lines or bronchial epithelial cells obtained by bronchoscopy, have also shown that corticosteroids have either a limited or no effect on inflammatory mediator release [37–39].

In the combination experiments, we frequently observed an additive effect, and the statistical analysis demonstrated a synergistic interaction between the corticosteroid and p38 MAPK inhibitor for some experimental conditions. The use of full dose-response curves allowed analysis of efficacy enhancement and dose-sparing effects to uncover these synergistic effects. This concurs with our previous data in alveolar macrophages from COPD and asthma patients [8, 11] and in bronchial epithelial cells [38], showing synergy using full dose-response curves.

An additive anti-inflammatory effect was observed with combination treatment in bronchial epithelial cells obtained by bronchoscopy. The lower cell number availability compared with using a cell line meant that we were unable to construct full dose-response curves, which represent the optimum way to analyse for additive or synergistic interactions [8, 11]. This analysis was performed on samples from asthma patients pooled with healthy controls to give additional statistical power. We observed no differences in the drug effects between the two groups, although it has been shown that other cell types from asthma patients can be corticosteroid resistant [19, 21, 22]. It would also be interesting to assess the effects of a p38 MAPK inhibitor on cells from patients with asthma who have been treated with ICS compared to steroid naïve asthmatics or healthy controls. The dexamethasone and BIRB-796 concentration used in the primary cells (100 nM) was selected as it was a suboptimal concentration in HBEC experiments on cytokine suppression, thus allowing possible pharmacological addition and synergy to be studied in combination experiments. The primary cells appeared to be less corticosteroid sensitive compared to HBECs, supporting our decision to use a relatively high but suboptimal concentration. Nevertheless, these experiments using cells from asthma patients confirm the potential anti-inflammatory advantages of combining these drug classes, with an additive effect observed on IL-6 production.

While in the present study all inflammatory mediators measured were reduced by glucocorticoids, other studies have shown increased expression of many inflammatory genes such as TLR2 or interferon regulatory factor 1 (IRF1) and CXCL10 [40, 41]. Glucocorticoids increase the expression of dual-specificity phosphatase-1 (DUSP1) which targets c-Jun N-terminal kinase (JNK), extracellular-regulated kinase (ERK) and p38 MAPK pathways induced by inflammatory stimuli to provide feedback inhibition [42]. Glucocorticoids decrease the phosphorylation and activation of p38 MAPK via DUSP1-dependent regulation in macrophages and epithelial cells [43]. The induction of IRF1 and consequently CXCL10 by glucocorticoids is mediated by the increase of DUSP1 and therefore reduced MAPK activity [41]. In the context of such systems where MAPKs are involved in feedforward regulation, it has been suggested that reduced repression of MAPKs could provide superior repression of inflammatory genes [44].

To further understand the mechanisms of the combination effects observed, we sought to investigate the role p38 MAPK plays on GR phosphorylation at various sites. GR phosphorylation at S211 has an important role in the transcriptional activity of GR [45], by potentially inducing a functionally active folded conformation [46]. A lack of S211 phosphorylation has been linked to glucocorticoid resistance in human lymphoid cells [47]. However, Chang et al. [9] found that GR nuclear localisation was reduced in airway smooth muscle cells from patients with severe asthma compared with healthy controls, but this reduction was not due to altered S211 phosphorylation. In lymphoid cells, S211 is a substrate for p38 MAPK to enhance GR to stimulate transcription [13]. However in alveolar macrophages, exposure to nontypeable *Haemophilus influenzae* (NTHi) causes p38 MAPK-dependent GR phosphorylation at S226 but not S211, resulting in decreased GR function [18]. The role of p38 MAPK activity in the regulation of GR transactivation has also been shown in airway smooth muscle cells via regulation of GR phosphorylation at S203 and S211 [15]. These data highlight potential differences in cell type-specific and stimuli-specific GR phosphorylation.

Phosphorylation of GR S226 is involved with shuttling of GR out of the nucleus [48]. While GR ligands increase phosphorylation of GR at both S211 and S226, it is the relative level of S211 versus S226 phosphorylation which is important. Comparatively, higher phosphorylation at S211 relative to S226 correlates with GR nuclear localization and greater transcriptional activity and vice versa [17]*.* We show that, in bronchial epithelial cells, the effect of p38 MAPK is to modulate S226 rather than S211 phosphorylation, which may lead to increased nuclear export of GR.

Our observations regarding GR S226 phosphorylation are supported by data from a study using PBMCs from severe asthma patients, whereby a reduction in GR nuclear translocation was associated with increased GR S226 phosphorylation compared with healthy controls [19]. Furthermore, IL-2 and IL-4 caused p38 MAPK-dependent phosphorylation of GR at S226 in a human monocytic cell line (U937 cells) [19]. Additionally, p38 MAPK inhibition has been shown to reduce phosphorylation of GR S226 induced by NTHi in alveolar macrophages [18] or by IL-2/IL-4 in a myeloid cell line [19].

In summary, we have shown molecular crosstalk between p38 MAPK activation and GR function in human bronchial epithelial cells. p38 MAPK inhibitors used in combination with corticosteroids are known to have additive anti-inflammatory effects [8, 11], and we show here the potential for synergistic effects on cytokine production from bronchial epithelial cells. Combining corticosteroids and a p38 MAPK inhibitor may be an effective treatment option in patients with moderate-to-severe asthma, where there is evidence of increased p38 MAPK activation.

## Electronic supplementary material

ESM 1 (PDF 482 kb)

## Abbreviations

ANOVA: Analysis of variance
ACQ: Asthma control questionnaire
CXCL: The chemokine (C-X-C motif) ligand
DUSP1: Dual-specificity phosphatase-1
ELISA: Enzyme-linked immunosorbent assays
ERK: Extracellular-regulated kinase
FEV_1_: Forced expiratory volume in 1 s
FKBP5: FK506-binding protein 51
GILZ: Glucocorticoid-induced leucine zipper
GINA: Global Initiative for Asthma
GR: Glucocorticoid receptor
HNS: Healthy never smoker controls
HBEC: Human bronchial epithelial cell
ICS: Inhaled corticosteroids
IL: Interleukin
IR: Interaction ratio
IRF1: Interferon regulatory factor 1
JNK: c-Jun N-terminal kinase
LPS: Lipopolysaccharide
MAPK: Mitogen-activated protein kinases
MEME: Minimal Essential Medium Eagle
NTHi: Nontypeable *Haemophilus influenzae*
PBMCs: Peripheral blood mononuclear cells
poly I:C: Polyinosinic:polycytidylic acid
RANTES: Regulated on Activation, Normal T Cell Expressed and Secreted
S: Serine
TNFα: Tumour necrosis factor
