# Supplementary material for: P38 MAPK and glucocorticoid receptor crosstalk in bronchial epithelial cells
Source: J Mol Med (Berl). 2020 Jan 23;98(3):361–74. doi: 10.1007/s00109-020-01873-3 (PMC7080672; doi:10.1007/s00109-020-01873-3)
Supplement: Supplementary file 1 — (PDF 482 kb) [file 109_2020_1873_MOESM1_ESM.pdf]

Title: P38 MAPK and glucocorticoid receptor cross-talk in bronchial epithelial cells

Authors: Simon Lea (1), Jian Li (1), Jonathan Plumb (1), Kate Gaffey (1), Sarah Mason (1), Rosie Gaskell (1), Chris Harbron (2) and Dave Singh (1).

Online Supplement

Methods

Cellular fractionation

HBEC were harvested and washed with PBS. Cells were lysed and separated into cytoplasmic and nuclear extracts by using NE-PER kit (NE-PER Nuclear and Cytoplasmic Extraction Reagents, Thermo Scientific). 100ul of CER I was added to cells and vortexed vigorously 15 seconds, then incubated on ice 10 minutes. 5.5ul of ice cold CER II was added into the lysates, vortexed 5 seconds twice followed by 5minutes maximum speed spin. Supernatants were transferred into new tubes as cytoplasmic extraction. Insoluble pellet which contains nuclei were suspended into 50ul NER buffer, followed by vortexing for 15 seconds every 10 minutes for a total of 40 minutes. Nuclear lysates were centrifuged at maximum speed for 10 minutes. Immediately the supernatants (nuclear extract) were transfered into new tubes.

Western blot analysis

Cells were lysed in RIPA buffer [10 mM Tris-HCl, pH 7.4, 150 mM NaCl, 1 mM EDTA, 1% Nonidet P-40, 0.25%] containing phosphatase (Sigma Aldrich, Dorset, UK) and protease inhibitors (Calbiochem, Nottingham, UK). Cell lysates diluted in sample buffer [62.5 mM Tris, 10% glycerol, 1% SDS, 1% β-mercaptoethanol, and 0.01% bromphenol blue, pH 6.8] were electrophoresed on SDS-polyacrylamide gels (10%) and transferred to Hy-bond ECL membranes (Whatman, Kent, UK).

Membranes were incubated with blocking buffer [5% dried milk in Tris-buffered saline containing 0.1% Tween 20 (TBS/Tween 20)] for 4 h at room temperature and then incubated with primary antibodies (diluted in blocking buffer) at 4 °C overnight. After washing in TBS/Tween 20, the membranes were incubated for 60 min with a peroxidase-conjugated secondary antibody (diluted in wash buffer), washed again, and the antibody-labeled proteins were visualized by enhanced chemiluminescence (Amersham Biosciences, Buckinghamshire, UK). Densitometric analysis was performed by normalising band density to that for total p38, total GR, β-actin or histone-3 using Quantity One v4.6.1 software (Bio-Rad, Hertfordshire, UK).

Stable transfection of 16HBE14o- cells

16HBE14o- cells were maintained in MEM (Invitrogen) supplemented with 10%FBS, penicillin/streptomycin and L-glutamine at 37°C in a humidified 5% CO_2 environment. For stable transfection, cells were cultured in 10-cm dishes at a density of 2 x 10^5. 6 μg of pGL44.36[luc2P/MMTV/Hygro] vector (Promega) (SalI linearized) were mixed with 18 μl of FuGENE6 and transfected into cells according to the manufacturer's manual (Roche, Germany). Positive colonies were selected for 3-5 weeks in the presence of Hygromycin (200 μg/ml). Individual colonies were isolated with sterile cloning discs (Sigma, UK) and transferred to 24-well plates until ready for the following experiments.

Bioluminescence Recording

Cells with luciferase reporters were seeded into 35 mm dishes with recording media that contains luciferin and ATP. Bioluminescence emissions from cell culture dishes were recorded in real time by Lumicycle (Actimetrics, USA). For drug treatment, individual dishes of cells were treated with dexamethasone, BIRB-796 and TNFα, either individually, or in combination, which were left continuously with the samples thereafter. Bioluminescence signals were analysed in BRASS software and RAP algorithm. Data were presented as photon counts per minute (cpm).

Real time qPCR

Culture supernatants were removed and cells were lysed in RLT buffer. Total RNA was purified from cell lysates using RNeasy kits (Qiagen, Crawley, UK) according to manufacturer's instructions. DNA contamination was prevented by on-column addition of DNase (Qiagen, Crawley, UK) according to manufacturer's instructions. Reverse transcription was performed on 50 ng of RNA using the Verso cDNA kit (Thermo Scientific). The resulting cDNA was reacted with ABsolute blue qPCR mix (Thermo Scientific) in 25 µl reactions containing premade ABI Taqman gene expression assays for GILZ (Hs00608272_m1), FKBP5 (Hs00188025_m1) and the endogenous control was glyceraldehyde-3phosphate dehydrogenase (GAPDH) (Catalogue no: 4352934E) (Applied Biosystems, Warrington, UK). Controls without RT-enzyme showed there was no genomic DNA amplification. Thermal cycling was carried out on a Stratagene MX3005P (Agilent Technologies, West Lothian, UK). Relative expression levels were determined using the $2^{-\Delta\Delta Ct}$ (untreated vs treatment).

IHC methods

Bronchial biopsies were fixed in 10% neutral buffered formalin for 8 hours and were processed using an automated tissue processing machine on a routine overnight schedule. Biopsies were embedded in histological grade paraffin wax and 3µm sections were cut with a Leica RM2235 rotary microtome. For immunohistochemical staining, slides were dewaxed using xylene and dehydrated through a series of industrial denatured alcohols. Heat-induced epitope retrieval was achieved using citrate buffer (pH 6.0) and a microwave. Staining was carried out with rabbit anti-human glucocorticoid receptor Ser226 (Abcam, ab93104; 1:150 overnight at 4^oC) and rabbit anti-human phosphorylated p38 MAPK (Cell Signaling Technology, #9211; 1:400 overnight at 4^oC) coupled with an ImmPRESS™ Excel Amplified HRP Polymer Staining Kit (Anti-Rabbit IgG) with 3,3' diaminobenzidine as a substrate (Vector, MP-7601). Sections were counterstained in Gills haematoxylin. A rabbit igG isotype, diluted to the same concentration as the relevant primary antibody was used as a negative control (Cell Signaling Technology, #3900).

Images of the stained slides were captured using a Nikon Eclipse 80i microscope (Nikon UK Ltd) with an attached QImaging digital camera (Media Cybernetics). The percentages of glucocorticoid receptor Ser226 and phosphorylated p38 MAPK positive cells were calculated using the cell counting tool in ImageJ (version 1.49, NIH). Numbers of positively- and negatively-stained cells were counted from the epithelium of each biopsy and the number of positively-stained cells was expressed as a percentage of the total number of epithelial cells.

Data analysis

Two analyses were performed to assess whether a combination of dexamethasone and BIRB-796 exhibited synergy: a dose-sparing analysis to assess whether equivalent responses can be achieved at lower doses of compound than expected given the monotherapy response of the two compounds, and an efficacy-enhancing analysis to assess whether the combination results in a significantly greater maximal effect than either compound alone as monotherapies. The dose-sparing analysis calculates a combination index with confidence intervals using the method described by (Harbron, 2010). The efficacy-enhancing analysis fits Hill dose-response curves to the monotherapy and combination results using both common and separate parameters for maximal response and tests for the improvement in fit from allowing the parameter to vary by using an F test. Both analyses were performed assuming a slope parameter in the Hill dose-response equation equal to one. Robustness analyses were also performed estimating the slope parameters and found to give the same conclusions.

Results

Inhibition of cytokine production from HBECs

The maximal inhibition of LPS and TNFα stimulated RANTES (47% and 49% respectively) and CXCL8 (63% and 62% respectively) secretion were lower compared with IL-6 (81% and 79%; $p<0.05$ for all comparisons) (Supplementary Table 1). The maximal inhibition of poly I:C stimulated IL-6, CXCL8 and RANTES secretion was similar ($p>0.05$ all comparisons).

The maximal inhibition of poly I:C stimulated IL-6, CXCL8 and RANTES secretion was similar ($p>0.05$); maximal inhibition of LPS stimulated cytokine production was also similar between cytokines. BIRB-796 caused significantly greater maximal inhibition of TNFα induced IL-6 compared to CXCL8 and RANTES ($p=0.02$ for both comparisons) and greater inhibition of TNFα induced CXCL8 compared to RANTES ($p=0.02$) (Supplementary Table 1).

There were no significant differences in levels of inhibition between dexamethasone and BIRB-796 at any concentration for cytokines induced by poly I:C (Figure 1). The inhibition of TNFα induced CXCL8 or RANTES were similar for dexamethasone and BIRB-796 at all concentrations, while the effect of dexamethasone on TNFα induced IL-6 was significantly greater compared to BIRB-796 at 10-1000 nM ($p<0.05$ for all comparisons). The inhibition of LPS induced CXCL8 or RANTES was similar at all concentrations, while the effect of dexamethasone on IL-6 was significantly greater compared to BIRB-796 at 1-1000 nM ($p<0.05$ for all comparisons).

Effect of p38 MAPK inhibition on TNFα-induced phosphorylation of p38 MAPK and GR at S226 and S211

In additional experiments, TNFα alone and in combination with dexamethasone showed increased p38 MAPK phosphorylation which was reduced by BIRB-796 (Supplementary Fig.5), while dexamethasone increased GR 211 phosphorylation which was not effected by TNFα and/or BIRB-796 treatment (Supplementary Fig.5); due to the small sample size (n=4 per condition), these changes were not statistically significant. Although TNFα alone and in

combination with dexamethasone showed a trend of increased GR 226 phosphorylation, this was not affected by the addition of BIRB-796 (Supplementary Fig.5).

Supplementary Table 1

Maximal inhibition dexamethasone (1000 nM)				
	Stimulant			pValue
	LPS	polyI:C	TNFα	
IL-6	81	65	79	0.39
CXCL8	63*	75	62*	0.068
RANTES	47*	69	48*	0.13
p Value	0.049	0.63	0.004	
**Maximal inhibition BIRB-796 (1000 nM)**				
	Stimulant			pValue
	LPS	polyI:C	TNFα	
IL-6	48	69	62	0.14
CXCL8	49	58	55*	0.63
RANTES	33	64	29*#	0.07
p Value	0.32	0.55	0.005	

*** = significantly lower than maximal inhibition of IL-6 ($p<0.05$)**

# = significantly lower than maximal inhibition of CXCL8 ($p<0.05$)

Supplemental Table 2

	**LPS**						
	**Dex**	**BIRB (1000nM)**	**Dex + BIRB (0.1nM)**	**Dex + BIRB (1nM)**	**Dex + BIRB (10nM)**	**Dex + BIRB (100nM)**	**Dex + BIRB (1000nM)**
**IL-6**	72	56*	78 #	78 #	83* #	89* #	88* #
**CXCL8**	69	64	69	77 #	78* #	86* #	85* #
**RANTES**	45	37	40	47	51 #	53 #	56 #
	**poly I:C**						
**IL-6**	72	62	68	73	77 #	84 #	85 #
**CXCL8**	73	72	71	79	76	87 #	85
**RANTES**	38	20*	37 #	46 #	54* #	55* #	56* #
	**TNFα**						
**IL-6**	80	78	79	82	86	89* #	90* #
**CXCL8**	56	70*	62	67*	71*	77*	79* #
**RANTES**	52	34*	26*	31*	39	36*	45

*** = significantly different compared to dexamethasone 1000nM alone (p<0.05)**

# = significantly different compared to BIRB-796 1000nM alone (p<0.05)

Percentage inhibition of IL-6			
	**HNS**	**Asthma**	**p- value**
**Dex**	13 ± 16	13 ± 9	0.99
**Birb**	31 ± 36	34 ± 16	0.84
**Dex + Birb**	50 ± 22	51 ± 26	0.99
**Percentage inhibition of CXCL8**			
	**HNS**	**Asthma**	**p- value**
**Dex**	36 ± 44	38 ± 15	0.95
**Birb**	53 ± 41	51 ± 11	0.94
**Dex + Birb**	59 ± 43	48 ± 17	0.67

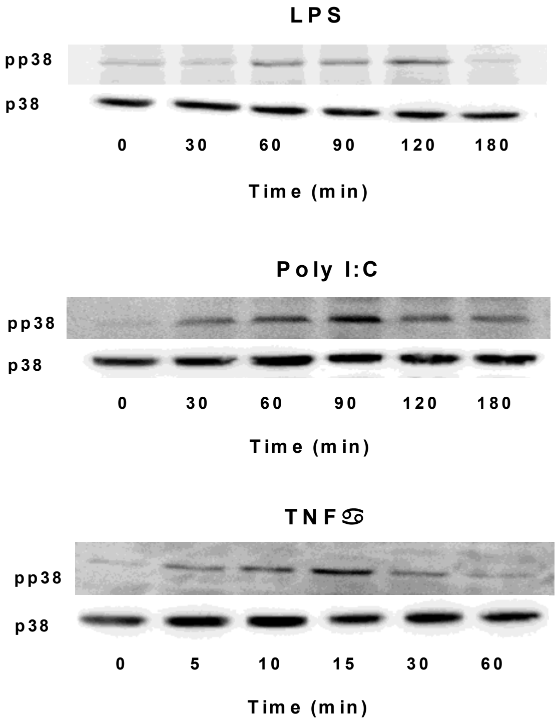

Supplement Fig.1: Effect of LPS, poly I:C and TNFα on phosphorylation of p38 MAPK

16HBE cells were stimulated with either LPS (1ug/ml) (A), poly I:C (100 µg/ml) (B) or TNFα (10 ng/ml) (C) for 15, 30, 60, 120 and 180 minutes. Cells were lysed and assessed for phosphorylation of p38 MAPK by Western blotting. Representative blots are shown (n=4).

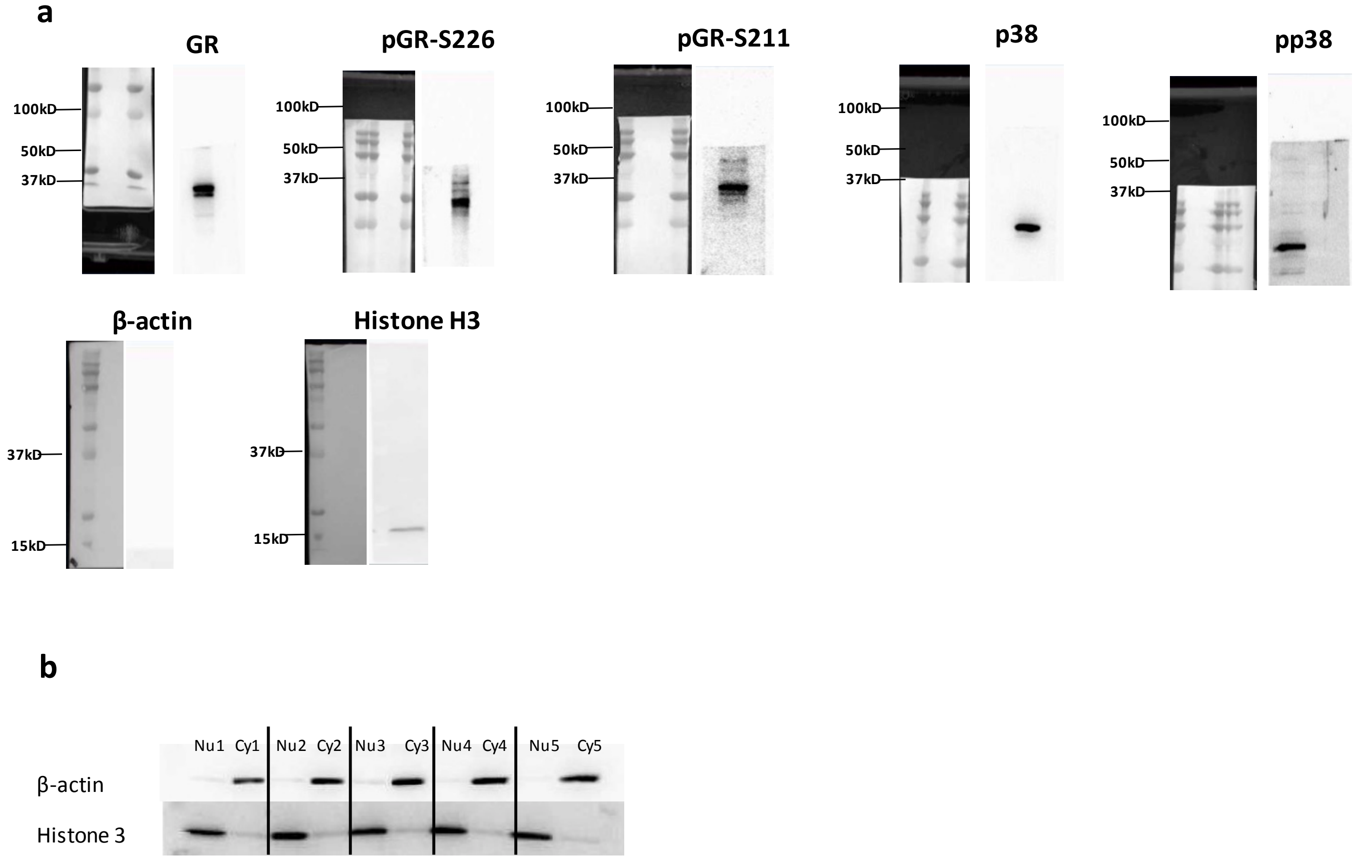

Supplement Fig.2: Western blot protein molecular weights and presence of nuclear and cytoplasmic proteins in cellular fractions

Untreated 16HBE cells were lysed (A) and underwent cellular fractionation (B). Protein size ladder was run alongside Western blot analysis for GR, pGR-226, pGR-211, p38, phosphor-p38, β-actin and histone-3 (A). The presence of nuclear protein histone-3 and cytoplasmic protein β -actin in nuclear (Nu) and cytoplasmic (Cy) fractions were determined by Western blot analysis (B). Images represents protein ladder and Western blot for same gel run (A). Image represent samples from n=5 separate cultures (B).

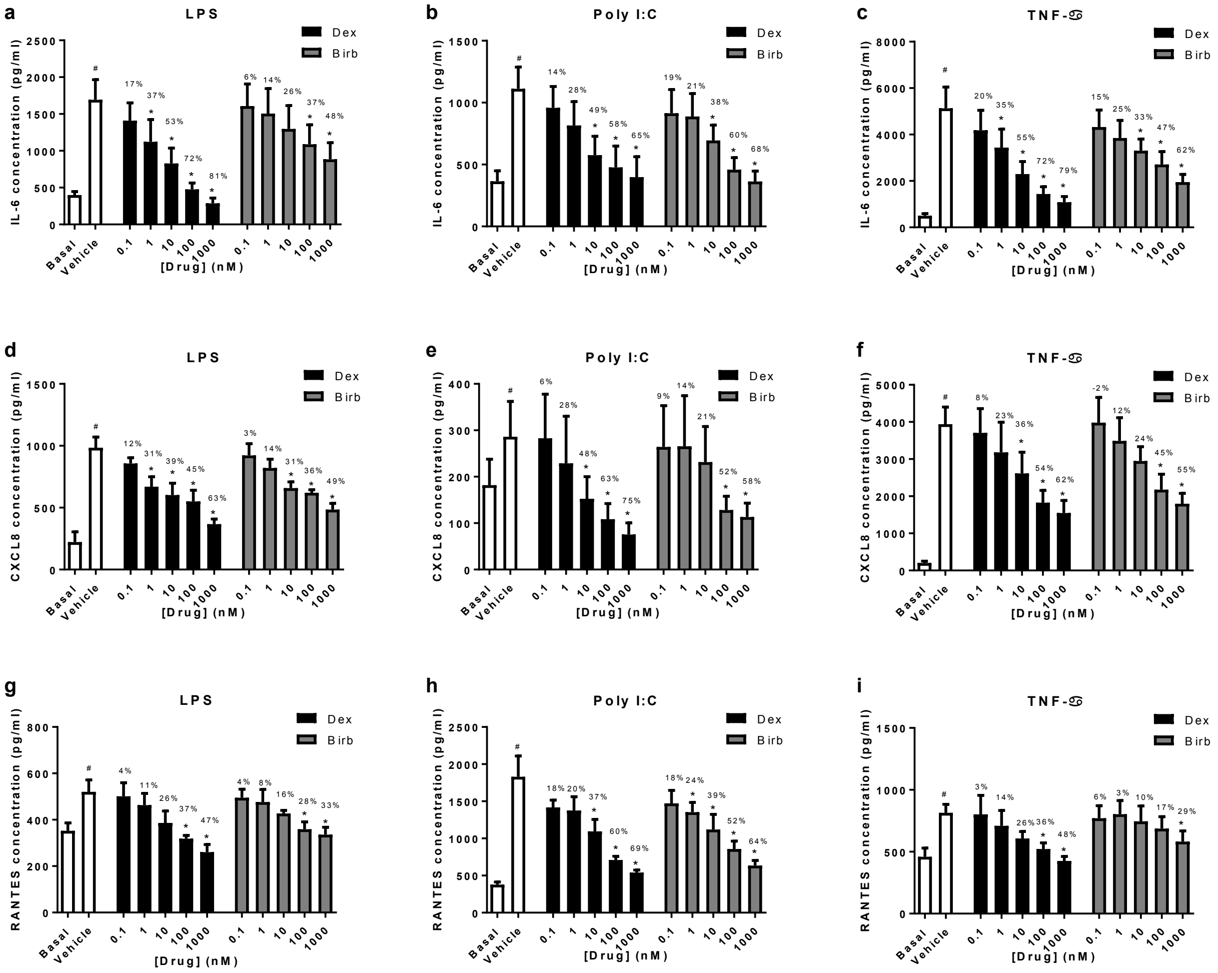

Supplement Fig.3: Effect of dexamethasone and BIRB-796 on LPS-, poly I:C- or TNFα-induced cytokines in human bronchial epithelial cells.

16HBE cells were pre-treated with dexamethasone (0.1-1000 nM), BIRB-796 (0.1-1000nM) or vehicle (DMSO 0.05%) for 1 hour before 24-hour stimulation with either LPS (1ug/ml), poly I:C (100 µg/ml), TNFα (10 ng/ml) or media (Basal). Supernatants were collected and assayed for IL-6 (A-C), CXCL8 (D-F) or RANTES (G-I) release by ELISA. Data presented as Mean±SEM of n=12 separate cultures with percentage inhibition in text above each bar.

# = significantly above basal levels ($p<0.05$)

* = significantly below vehicle control ($p<0.05$)

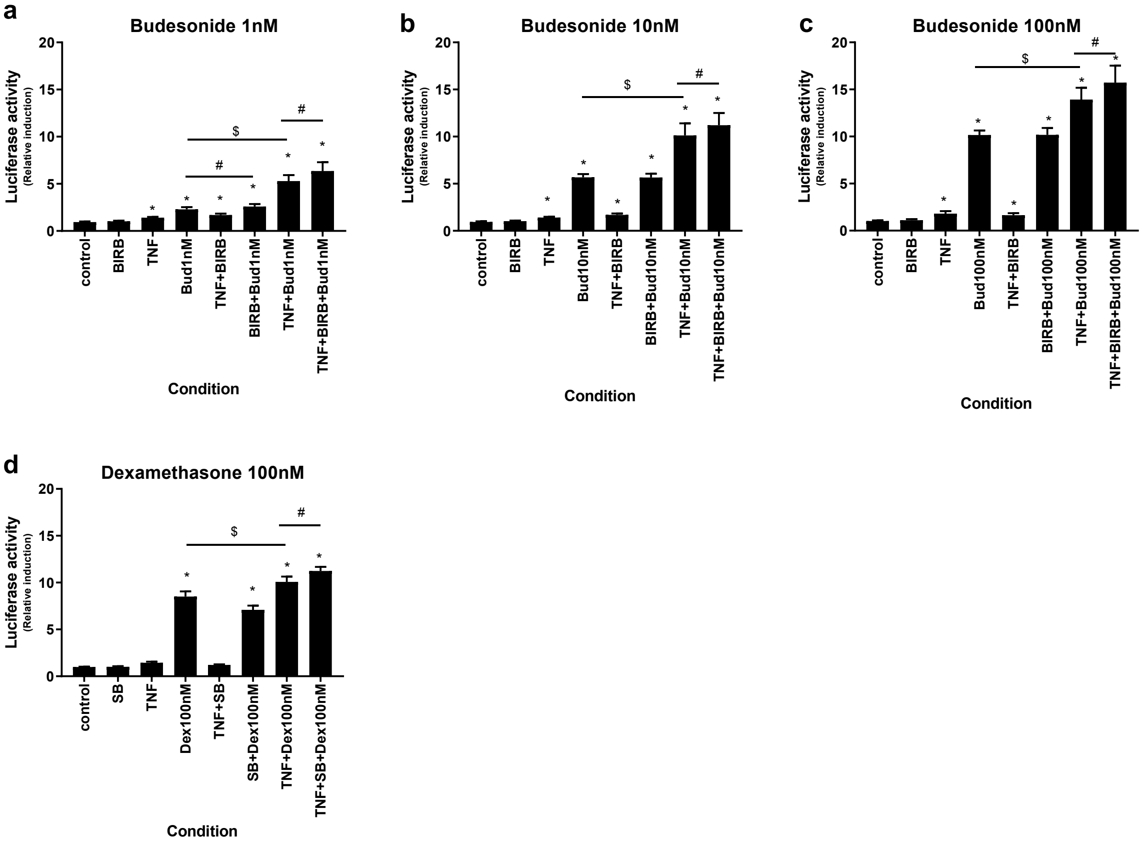

Supplement Fig.4: Effects of p38 MAPK inhibition on budesonide and dexamethasone induced GR reporter activity

16HBE cells transfected with GR-luciferase reporter were stimulated with TNF-α (10ng/ml) or left unstimulated followed by treatment with budesonide (1-100 nM) (A-C) or dexamethasone (100nM) (D), BIRB-796 (1000 nM) alone and in combination with budesonide at all concentrations or SB239063 (1000 nM) alone and in combination with dexamethasone (100 nM) (D). GR-reporter activity was assessed by luminescence. Data represent mean ± SEM of relative activity compared to basal levels from n=6 experiments.

* = significantly above basal control ($p<0.05$)

# = significantly increased above steroid alone in either stimulated or unstimulated cells ($p<0.05$)

$ = significantly increased above steroid alone in unstimulated cells ($p<0.05$).

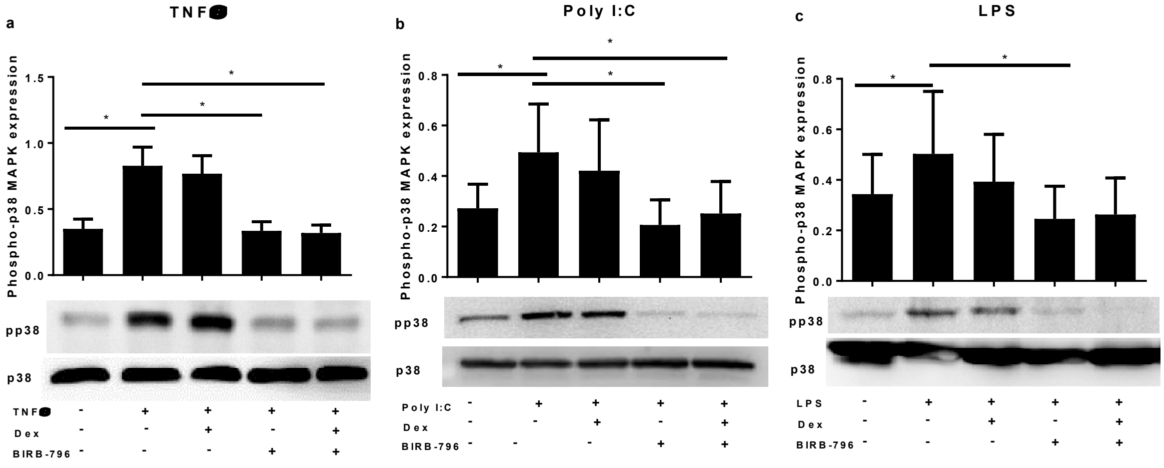

Supplement Fig.5: Effect of dexamethasone and BIRB-796 on LPS, poly I:C or TNFα induced phosphorylation of p38 MAPK

16HBE cells were stimulated with TNFα (10 ng/ml) (A), poly I:C (100 μg/ml) (B) or LPS (1μg/ml) (C) for 30 minutes with or without 30 minutes pre-treatment with dexamethasone (1000nM) or BIRB-796 (1000nM) alone or in combination. Cells were lysed and assessed for phosphorylation of p38 MAPK by Western blotting. Band density was normalised to total p38. Representative blots are shown under corresponding conditions. Data presented as Mean±SEM (n=5).

* = significant difference between condition ($p<0.05$).

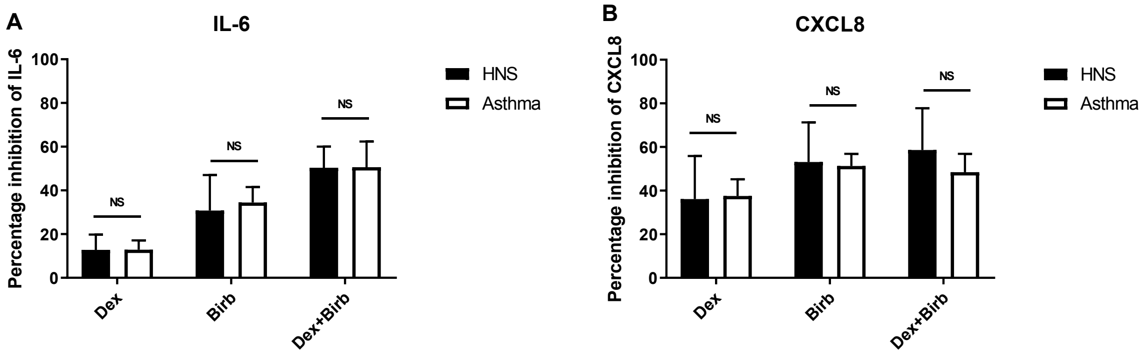

Supplement Fig.6: Combination effect of dexamethasone and BIRB-796 on TNFα-induced cytokines in primary human bronchial epithelial cells.

Primary bronchial epithelial cells from healthy subjects (HNS) (n=5) or patients with asthma (n=5) were pre-treated with dexamethasone (100 nM) and BIRB-796 (100 nM) alone or in combination or with vehicle (DMSO 0.05%) for 1 hour before 24-hour stimulation with TNFα (10 ng/ml) or media (Basal). Supernatants were collected and assayed for IL-6 (A) or CXCL8 (B) release by ELISA. Data presented as Mean±SEM percentage inhibition of LPS induced cytokine levels. 2way ANOVA followed by Bonfferoni's post analysis used to compare between HNS and Asthma for all conditions.

HARBRON, C. 2010. A flexible unified approach to the analysis of pre-clinical combination studies. *Stat Med,* 29**,** 1746-56.
